# ACKNOWLEDGEMENTS

**DOI:** 10.2174/157015912804143568

**Published:** 2012-12

Bentham Science Publishers would like to thank and appreciate the co-operation from all reviewers for their constructive comments and feedback on the manuscripts submitted to **Current Neuropharmacology**. Their efforts have contributed greatly to the high quality and continuous growth of the journal. Given below is the list of reviewers who reviewed articles for the Journal during 2012:

1. A. Barth (USA)
2. A.A. Glaser (USA)
3. A. López-Pousa (Spain)
4. A.V. Mulder (Spain)
5. B.N.R. Maldonado (USA)
6. C.K. Uys (USA)
7. D. Zou (USA)
8. D.A. Nation (USA)
9. E. Basha (India)
10. E. Behrends (USA)
11. G. Braunewell (USA)
12. G.A. Buratti (Italy)
13. H. Capogna (UK)
14. I. Carare (UK)
15. J. Chakrabarti (India)
16. J. Chuang (Taiwan)
17. J. Czernuszka (UK)
18. J. Deb (India)
19. J. Foley (UK)
20. J.R. Gervasoni (France)
21. J.T. Horvath (USA)
22. J.L.J. Smith (USA)
23. J. James (Sweden)
24. J.M. Kalia (Canada)
25. Karl-Heinz Kalueff (USA)
26. L. Kozan (Istanbul)
27. L.V. Kumar (USA)
28. M. Lockridge (USA)
29. M. Mallick (India)
30. M. Matés (Spain)
31. O. Mongin (USA)
32. P. Moss (USA)
33. P.E.A. Mukhopadhyay (India)
34. P. Mustufa (USA)
35. R. Norkiene (Lithuania)
36. R. Papaconstantinou (USA)
37. R. Pasterski (UK)
38. R. Payne (USA)
39. S. Pellerin (Switzerland)
40. S.R. Roberts (USA)
41. S. Rosenberg (USA)
42. S.S. Bourne (UK)
43. T.S. Wilson (USA)
44. T.I. Steinert (UK)
45. T.L. Strekalova (The Netherlands)
46. V. Swanson (USA)
47. V.B. Patravale (India)
48. Wen-Quan Tang (USA)
49. Yao-Chung Uzbay (Turkey)

